# Fractal Laws for Bifurcation Quantitative Coronary Angiography to Assess Left Main Bifurcation Lesions

**DOI:** 10.1155/crp/7176161

**Published:** 2025-06-01

**Authors:** Mattia Lunardi, Nozomi Kotoku, Carlo Briguori, Luc Maillard, Adam Kern, Franck Digne, Jacek Legutko, Maciej Lesiak, Adam Witkowski, Thierry Lefèvre, Anderzej Ochala, Wojciecj Jachec, Corrado Tamburino, Marco Contarini, Gilles Rioufol, Antonio Colombo, Javier Escaned, William Wijns, Yoshinobu Onuma, Patrick W. Serruys, Robert Gil

**Affiliations:** ^1^Department of Cardiology, University of Galway, Galway, Ireland; ^2^The Lambe Institute for Translational Medicine and CURAM, University of Galway, Galway, Ireland; ^3^Interventional Cardiology Unit, Mediterranea Cardiocentro, Naples, Italy; ^4^GCS ES Axium Rambot, Aix en Provence, France; ^5^Department of Cardiology and Internal Medicine, Medical Faculty, University of Warmia and Mazury in Olsztyn, Olsztyn, Poland; ^6^Centre Cardiologique du Nord, 32-36 Rue des Moulins Gémeaux, Saint-Denis 93200, France; ^7^Department of Interventional Cardiology, Institute of Cardiology, Jagiellonian University Medical College, Krakow, Poland; ^8^Clinical Department of Interventional Cardiology, The John Paul II Hospital, Krakow, Poland; ^9^Department of Cardiology, Poznan University of Medical Sciences, Poznan, Poland; ^10^Department of Interventional Cardiology and Angiology, National Institute of Cardiology, Warsaw, Poland; ^11^Institut Cardiovasculaire Paris Sud, Hospitalier Privé Jacques Cartier, Ramsay Générale de Santé, Massy, France; ^12^Department of Cardiology, Gornoslaskie Centrum Medycnze, 45/47, Katowice 40-635, Poland; ^13^2nd Department of Cardiology, Faculty of Medical Sciences in Zabrze, Medical University of Silesia, Katowice, Poland; ^14^Division of Cardiology, CAST, P.O. “Rodolico”, Azienda Ospedaliero-Universitaria, “Policlinico-Vittorio Emanuele”, University of Catania, Catania 95123, Italy; ^15^Cardiology Department, Umberto I Hospital, ASP 8 Siracusa, Syracuse, Italy; ^16^Cardiology Department, Hospices Civils de Lyon & Carmen, Lyon, France; ^17^Department of Biomedical Sciences, Humanitas University, Pieve Emanuele, Milan, Italy; ^18^Humanitas Research Hospital IRCCS, Rozzano, Milan, Italy; ^19^Department of Cardiology, Hospital Clínico San Carlos IDISCC, Complutense University of Madrid, Madrid, Spain; ^20^Department of Cardiology, Central Clinical Hospital of the Internal and Administration Ministry, Warsaw, Poland

**Keywords:** bifurcation, iFR, left main, QCA

## Abstract

**Background:** Visual angiographic assessment of left main (LM) bifurcation lesions is fraught with major limitations. Bifurcation-dedicated quantitative coronary angiography (Bif-QCA) assessment provides higher accuracy than standard QCA in bifurcation lesions. Fractal laws (e.g., Finet's and Murray's laws) can enhance the accuracy of reference diameter calculation when applied to angiography-derived algorithms and may serve as a surrogate for pressure-based assessment.

**Aims:** To investigate the correlation between Bif-QCA, Finet's law derived Bif-QCA (Finet-QCA) and pressure–wire functional assessment for LM bifurcation stenosis.

**Methods:** Using instantaneous wave-free ratio (iFR) as a reference standard (≤ 0.89), we compared the value of Bif-QCA and Finet-QCA (diameter stenosis ≥ 50%). Moreover, the differences in MEDINA classification according to site-reported visual assessment *vs* Bif-QCA or Finet-QCA were investigated.

**Results:** Eighty-four patients were included in the analysis, of which 72 (85.7%) presented an abnormal iFR. Bif-QCA derived %DS was moderately correlated with iFR values; however, implementing Finet's law in the correlation resulted weak. Site-reported MEDINA (visual assessment) resulted in significant higher rate of 1,1,1 and lower rate of 1,0,0 patterns compared to Bif-QCA MEDINA (9.5% vs. 1.2%, *p* < 0.001 and 33.3% vs. 46.4%, *p* < 0.001, respectively) and to Finet-QCA MEDINA (9.5% vs. 2.4%, *p* < 0.001 and 33.3% vs. 40%, *p* < 0.001, respectively).

**Conclusions:** The present study suggested that LM MEDINA bifurcation pattern should be based on QCA analysis rather than visual assessment, both in the context of clinical practice and clinical studies. Compared to conventional Bif-QCA, the implementation of fractal laws (Finet-QCA) did not appear to improve the determination of the reference diameters of the LM shaft.

## 1. Introduction

Percutaneous coronary intervention (PCI) of unprotected left main (LM) with low and intermediate SYNTAX score has been shown to be comparable in terms of major adverse cardiac and cerebrovascular events to coronary artery bypass graft (CABG) [1–4]. However, repeat revascularizations and spontaneous myocardial infarction (MI) are more common after PCI than after CABG. This risk is much higher in the presence of LM bifurcation stenosis, requiring complex stenting techniques [5].

Hence, along with the baseline characteristics, adequate grading of the number of narrowed branches part of the LM bifurcation, typically represented with the MEDINA classification, plays a key role in defining PCI strategies in LM bifurcation lesions, given the importance of avoiding unnecessary and potentially harmful interventions [6].

With this regard, the angiographic visual assessment on top of its subjective nature has major limitations in assessing the severity of LM stenosis and the distribution of the plaque between the main vessel and its daughter branches, due to multiple factors such as foreshortening, lack of any reference segment in short or diffusely diseased LM, or vessel-size mismatch between the proximal main trunk and the distal branches [7, 8]. Conventional single-vessel quantitative coronary angiography (QCA) software is inaccurate in bifurcation lesions because it completely ignores the natural anatomy of the bifurcation, including the natural “step-down” in diameters at the site of the bifurcation resulting in an inappropriate selection of reference diameters (RefDs) in computation of relative diameter stenosis (DS) [6, 9].

Consequently, bifurcation-dedicated QCA (Bif-QCA) software with segmental analysis has been proposed and provides higher accuracy for quantification of the degree of bifurcation stenosis [6, 10]. Furthermore, in some atherosclerosis patterns, such as diffuse LM atheroma, the proper calculation of RefDs is challenging, and the implementation of fractal laws (i.e., Finet et al. [11] and Murray's [12] laws) to QCA assessment may further improve its accuracy.

When possible, the treatment of LM bifurcation lesions should be based on their hemodynamic significance or intravascular appearance [13, 14].

**Figure 1:**
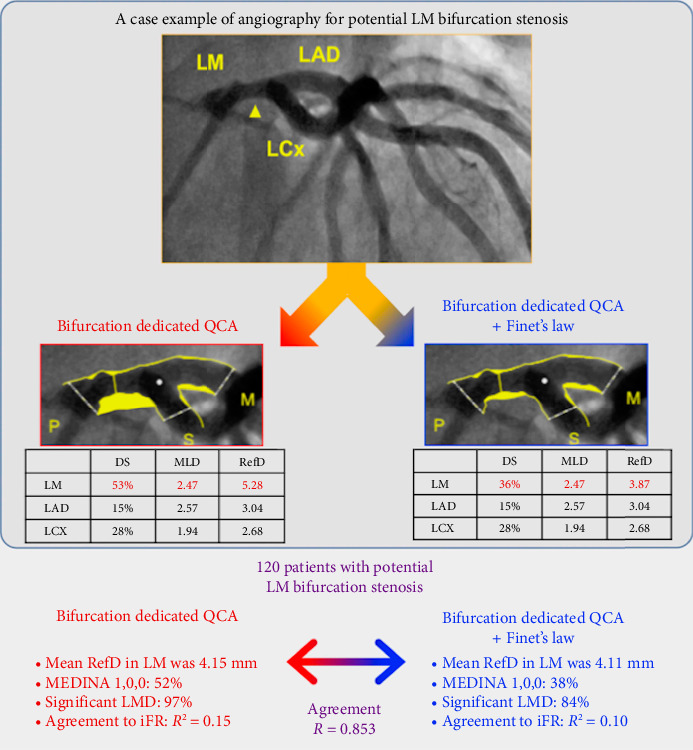
Central illustration: bifurcation dedicated quantitative coronary angiography (QCA) vs. Finet-derived bifurcation QCA.

The aim of the current study was to investigate the correlation between Bif-QCA, Finet's law derived Bif-QCA (Finet-QCA), and pressure-wire functional assessment for LM bifurcation stenosis (Figure 1).

## 2. Methods

### 2.1. Study Description

This is an observational study, including patients who underwent diagnostic angiograms revealing LM distal stenosis, with the exclusion of MEDINA 0,0,1 lesions. Included patients were part of the POLBOS LM [15] trial, a prospective, multicenter single arm study aiming at investigating the safety and efficacy of the BiOSS LIM C sirolimus-eluting cobalt chromium stent (Balton, Warsaw, Poland) in LM bifurcation lesions (Supporting Information).

Each patient signed the consent form prior to any study-specific assessment, confirming the patient's willingness to participate in the study. The informed consent process was performed in accordance with the requirements of the local ethics committee at each participating site (ethics committee approval number at the leading center is 47/2018 and 85/2018). The present analysis focuses on the diagnostic phase of the trial and does not report procedural or outcome data regarding the device under investigation.

The POLBOS LM sample size was calculated as 256 patients, but as a result of the COVID-19 pandemic, the study was put on hold and was not resumed. The sample size calculation was based on the hypothesis whereby 1-year clinical outcomes (patient oriented composite endpoint [POCE]) after BiOSS LIM C implantation in distal LM are not inferior to the best-in-class stent (XIENCE) used as the default stent in the EXCEL trial (POCE 16.7% at 12 months).

Eventually, this series includes the first 127 patients enrolled in the trial, of which 84 underwent invasive functional assessment and therefore deemed suitable for the present analysis.

### 2.2. Bif-QCA Fundamentals

RefD and %DS, along with the minimum lumen diameter (MLD), are computed for each coronary segment (proximal main vessel [PMV], distal main vessel [DMV], and side branch [SB]) by a Bif-QCA software (i.e., CAAS; Pie Medical Imaging, Maastricht, the Netherlands) that models the bifurcation contours after the definition of some reference points of the bifurcation by the analyst.

In specific settings, such as the presence of diffuse LM disease, the LM RefD derived from QCA is less accurate, given the absence of healthy segments where the software normally calculates the reference vessel dimensions, with obvious clinical implication.

To overcome this limitation, fractal laws can help to better estimate the PMV diameter from the two daughter-vessel diameters. Essentially, morphological self-similarity has been observed in coronary tree, and the sizes of the coronary segments of bifurcations have been demonstrated to be governed by fractal geometry [16]. In other words, the relation of the mother-vessel diameter (RefD_PMV_) and the two daughter-vessel diameters (RefD_DMV_ and RefD_SB_) in nature coronary tree holds at all levels of bifurcations: i.e., whatever diameter of the mother vessel. The relation has been described by the following bifurcation fractal laws.

1. The Murray's law [17]: RefD_PMV_^3^ = RefD_DMV_^3^ + RefD_SB_^3^
2. The Finet's law [11]: RefD_PMV_ = 0.678 × (RefD_DMV_ + RefD_SB_)
3. The Huo–Kassab's law [18]: RefD_PMV_^7/3^ = RefD_DMV_^7/3^ + RefD_SB_^7/3^

Accordingly, after QCA, the analyst implements into these formulas the RefD of the two daughter vessels to derive the LM RefD. Previous studies have demonstrated that Huo–Kassab's and Finet's laws had better correlation with fractional flow reserve (FFR) assessment [19].

### 2.3. LM Stenosis Severity Assessment

In the current study, LM bifurcation disease (%DS ≥ 50) was diagnosed first by visual assessment following coronary angiography and then confirmed by an independent academic research team (initially based in Rotterdam, the Netherlands, and subsequently relocated in Galway, Ireland) using the Bif-QCA software with and without Finet's law implementation.

Overall, LM bifurcation stenosis was defined according to different techniques.

1. Site-reported visual assessment: any distal LM DS ≥ 50% excluding the MEDINA class 0,0,1. Selection of the ideal angiographic view to optimize the profile of the distal LM carina geometry, maximally opening the bifurcation angle, was recommended.
2. Core laboratory-reported Bif-QCA with segmental analysis: MLD, RefD, and the %DS were reported for each vessel segment (LM, LAD, and LCx) [6, 20].
3. Finet's law-adjusted Bif-QCA (Finet-QCA): adjustment of the RefD of PMV as described above. Finet-derived RefD and %DS of LM were reported for all cases.
4. Instantaneous wave-free ratio (iFR) assessment: preprocedure iFR was left to operators' decision. The pressure wire (Verrata, Philips Volcano, Amsterdam, The Netherlands) was advanced distal to the target lesion either in left anterior descending artery (LAD, *n* = 83) or in left circumflex artery (LCx, *n* = 55) or in both (2 measurements (*n* = 54), followed by iFR pullback. The lowest iFR value was considered for statistical purposes.

### 2.4. Endpoints

The primary endpoint was to test the correlation between Bif-QCA, Finet-QCA, and pressure-wire functional assessment for LM bifurcation stenosis.

The secondary endpoints were as follows:

i. The difference in MEDINA classification according to site-reported visual assessment *vs* core-lab-reported QCA
ii. The impact on the respective enrollment eligibility according to Bif-QCA and Finet-QCA methods apart

### 2.5. Statistical Analysis

Continuous variables are presented as mean and standard deviation or as median and interquartile range depending on their distribution, whereas categorical variables are presented as frequencies (percentages). Examination of continuous variables normal distribution was performed with Kolmogorov–Smirnov test.

The extent of agreement between different techniques to assess the MEDINA classification beyond the level of chance was measured as a percentage of the total agreement using the kappa statistic. The kappa value was calculated using the standard formula: [observed agreement − expected agreement]/[1 − expected agreement].

QCA derived variables were examined by bivariate correlation analysis, and Pearson's correlation coefficients were obtained to determine if they have linear relation with iFR values.

All statistical analyses were performed using SPSS software, Version 25 (IBM Corp., Armonk, 281 N.Y., USA).

## 3. Results

### 3.1. Baseline Characteristics

Eighty-four patients were included in the present analysis, from 14 centers across Europe. Mean age of patients was 68.2 ± 9.4 years. Seventeen were female (20.2%). Baseline characteristics are listed in Supporting Table 1.

The most reported MEDINA classification, as per site visual estimation, was 1,0,0 (33.3%). True bifurcation lesions (1,1,1; 1,0,1; and 0,0,1) amounted to 18 (21.4%).

### 3.2. Bif-QCA Details

All cases underwent Bif-QCA, including Finet's law-derived measurements for LM segments (Finet-RefD and Finet-%DS) (Table 1).

In 39 cases (46.4%), the Finet-%DS resulted in greater values than the standard %DS (+7% [IQR +3%, +14%], *p* < 0.001); in 5 (5.9%), it was the same, and in the remaining 40 (47.6%), it was lower (−6% [IQR −2%, −9%], *p* < 0.001).

### 3.3. MEDINA Classification

The actual enrollment was based on the highest %DS according to Bif-QCA and Finet-QCA. Accordingly, the most frequent MEDINA was 1,0,0 (50%), followed by MEDINA 0,1,0 (27.4%), 1,1,0 (14.3%), 0,1,1 (3.5%), 1,0,1 (2.4%), and 1,1,1 (2.4%).

In comparison, the site reported MEDINA (based on visual assessment), significantly differed, resulting in slightly higher rates of MEDINA 1,1,1 (9.5% vs. 2.4%) and lower rates of 1,0,0 (33.3% vs. 50%, *p* < 0.001), showing a low level of agreement with QCA-based MEDINA (64%, kappa = 0.510) (Table 2). When compared with the 2 QCA methods apart, the level of agreement of visual assessment MEDINA was even lower (Tables 3 and 4).

Distribution of MEDINA classes according to the Bif-QCA and Finet-QCA methods apart is reported in Table 5. In particular, with Finet's law, MEDINA 1,0,0 decreased from 46.4% to 40.5%, with a consequent increase of MEDINA 0,0,0 (2% vs. 9.5%, *p* < 0.001). Overall, there was a moderate agreement between the two MEDINA classifications (78.6%, kappa = 0.676).

### 3.4. Impact of Different MEDINA Classifications on the Eligibility Rate

Patients' eligibility was reassessed by means of the two QCA methods apart. Based on the Bif-QCA, 3 fewer patients would have been included: 2 presenting MEDINA class 0,0,0 and 1 MEDINA class 0,0,1. In all these cases, the application of Finet's law resulted in LM DS% > 50, leading to classify such lesions as MEDINA class 1,0,0 and 1,0,1.

Conversely, on the basis of the Finet-QCA, 8 patients would have been excluded, presenting MEDINA classes 0,0,0, instead of 1,0,0.

Accordingly, the respective rate of significant bifurcation LM stenosis (MEDINA 0,0,1 excluded), compared to the actual enrollment, amounted to 97.6% (*n* = 82) and 90.5% (*n* = 76).

### 3.5. iFR Assessment

Out of 84 patients, 54 underwent iFR measurements placing the pressure wire both in LAD and LCx, 29 only in LAD (for a total of 83 LAD measurements), and 1 only in LCx (for a total of 55 LCx measurements). Overall, 72 (85.7%) presented an abnormal value (≤ 0.89). The median iFR value was 0.79 [IQR 0.69–0.90], considering the lowest value obtained either in LAD or in LCx when both were available. In these cases (*n* = 54), LAD iFR was positive in 50 cases (92.5%), while LCx iFR was positive in 19 cases (35.2%). Similarly, lower iFR values were more frequently derived from LAD measurement than in LCx (Supporting Table 2).

### 3.6. Correlation of Bif-QCA and Finet-QCA Variables With iFR

Bif-QCA derived %DS was moderately correlated with iFR (Pearson correlation coefficient of −0.402, *p* < 0.001).

When implementing the Finet's law to derive LM %DS, the correlation with iFR was weak, with a Pearson correlation coefficient of 0.326, *p*=0.02.

**Figure 2:**
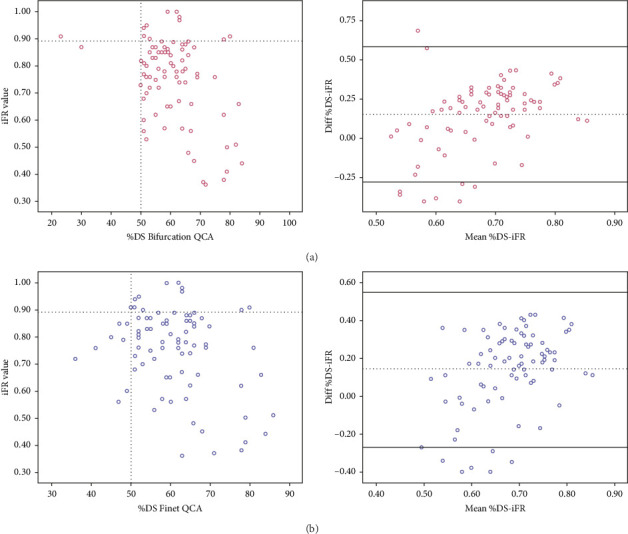
Correlation (left panels) and agreement (right panels) between iFR and Bif-QCA (a, top panels) and between iFR and Finet-QCA (b, bottom panels). (a) iFR vs. Bif-QCA: *R* = 0.402, *R*^2^ = 0.152, *p* < 0.001; (b) iFR vs. Finet-QCA: *R* = 0.326, *R*^2^ = 0.096, *p*=0.02.

Figure 2 shows the correlation and agreement between each technique and iFR. Supporting Figure 1 reports the related ROC curves.

## 4. Discussion

The present analysis investigated different angiography-based diagnostic approaches for the quantitative assessment of coronary lesions involving the LM bifurcation. The main findings can be summarized as follows:

1. The implementation of Finet's fractal law in the Bif-QCA algorithm did not improve the correlation with iFR, compared to the standard Bif-QCA.
2. Severity and MEDINA pattern of LM bifurcation stenosis significantly varied according to the assessment technique, with obvious implications on the treatment.

In this study, adjudications on eligibility and treatment decisions were taken according to the worst %DS calculated in the LM bifurcation on the basis either of the Bif-QCA or of the Finet-QCA. The rationale of such an approach lies in the fact that it has been argued that Bif-QCA software does not address comprehensively the complex physical interpretation of the reference vessel size at the bifurcation core, leading to inaccurate measurements [19]. Tu et al. recently proposed a dedicated bifurcation 3D-QCA method combined with different fractal laws (Murray's, Finet's, and Huo–Kassab's laws) and demonstrated that the application of the Huo–Kassab's and Finet's laws to calculate the %DS was associated with a better correlation with FFR measurements (*r* = −0.50 and −0.49, respectively, *p* < 0.001) than the Bif-QCA alone. However, the percentage of LM bifurcation stenosis in their series was relatively low (3 patients, 3.8%). Evidence related to the implementation of fractal laws in LM QCA is virtually inexistent. From a small series (73 patients) of LM stenting, Rigatelli et al. found that a Finet's law–guided stent sizing led to better cardiovascular outcomes at 5 years, compared to stent selection based on the standard QCA. Despite the attractive findings, the authors did not implement any Bif-QCA method in their analysis, limiting the validity of their results [21]. Another interesting comparison between standard angiography, standard QCA, Finet-derived QCA, and IVUS (55 patients) revealed that the Finet's law applied to the standard QCA unmasked 31% diffusely atherosclerotic LM (IVUS confirmation), otherwise considered normal by standard angiography and standard QCA [22]. Of note, even in this study, no Bif-QCA algorithms were used.

According to these studies, we decided to implement—among the three presented fractal laws—Finet's law to the standard QCA algorithm, aiming at increasing its diagnostic accuracy with subsequent potential better outcomes.

However, so far, there is no evidence on the added benefits of Finet's law to a Bif-QCA algorithm for LM stenosis, and some clinical scenarios might favor either standard algorithms, or modified ones (Supporting Figure 2).

First and foremost, our data confirmed that the mere visual estimation was inaccurate compared to QCA [23], leading to a higher lesion classification as MEDINA 1,1,1, therefore defining both LAD and LCx as severe. Although ostial disease is only one of the numerous parameters to consider an upfront double stent technique, operators may be prone to reconsider their strategy for the LM bifurcation revascularization, increasing the risk of short- and long-term PCI-related complications [24].

Second, the use of the two different QCA strategies apart led to changes in MEDINA classifications, which in turn would have translated into a reduction of the study eligibility rate, and therefore into more conservative treatments. However, this variation was substantial only using the Finet-QCA, which downgraded 8 lesions (9.5%), from MEDINA 1,0,0, to MEDINA 0,0,0.

Although Finet-QCA derived %DS values tended to be lower than those derived from Bif-QCA, it is not self-evident that the better is the most accurate. Along this line, we have investigated the correlation of each method against iFR.

iFR was recently reported to be safe to guide LM revascularization when using the standard cutoff of 0.89 [13]. Yet, in a retrospective registry of 125 LM stenosis, iFR of ≤ 0.89 correlates with IVUS minimum lumen area < 6 mm^2^, with an area under the curve of 0.77 (77% sensitivity and 66% specificity; *p* < 0.0001), supporting the use of iFR for the evaluation of intermediate LM stenosis [25].

We found that Bif-QCA derived LM %DS presented a moderate correlation with iFR values.

Interestingly, LM %DS, as calculated with Finet's fractal law, showed a weaker correlation with iFR values, suggesting less accuracy in identifying positive iFR lesions.

Of note, all lesions reported to be MEDINA 0,0,0 by Finet-QCA had an abnormal iFR.

However, the present analysis was not intended to test if such Bif-QCA methods can predict lesions producing ischemia as compared to iFR, and dedicated studies need to be performed.

Previous studies on single vessel lesions largely demonstrated the poor correlation between invasive functional assessment and QCA, with the latter identifying critically angiographic stenosis when FFR was normal in up to 20% of cases and viceversa [26]. In fact, vessel size is not the unique predictor of the amount of myocardium subtended by a given vessel; hence, the ischemic burden could not be limited to its quantification.

This is even more relevant in the case of bifurcations, where the complex coronary anatomy and the subtended amount of myocardium are hardly derivable from a pure quantitative analysis of the vessel dimensions. Hence, taking into account the data deriving from single vessel lesions, we may expect even a worse correlation between functional assessment and quantitative analysis of bifurcation lesions.

To overcome some of these limitations, however, bifurcation-dedicated software has been developed aiming to better estimate the original anatomy of such lesions. However, according to our results, they still present some limitations for the identification of critical lesions requiring revascularization.

Yet, looking at these data, we could assume fractal laws (i.e., Finet-QCA) may be good instruments for sizing optimization, more than for physiological assessment.

### 4.1. Limitations

We should consider the results as hypothesis generating and further assess the validity of these two angiographic methodologies against other established techniques (i.e., intravascular imaging). Currently, a coronary computed tomography angiography (CCTA), performed prior to the invasive fluoroscopic angiography, allows the selection of a so-called optimal 3D CCTA view, without overlapping of the two daughter branches, with visualization of both ostia, and with maximal angulation and minimal fore shortening [27]. The translation of this optimal 3D anatomic view into a 2D fluoroscopic view enhances considerably the feasibility and the concordance of the FFR–computed tomography with angiography-based functional assessment and Bif-QCA.

## 5. Conclusions

This study suggests that the LM bifurcation disease pattern should rely on QCA analysis rather than mere visual assessment. However, compared to conventional Bif-QCA, the application of fractal laws (e.g., Finet-QCA) does not appear to enhance the accuracy of determining the RefDs of the LM shaft.

## Figures and Tables

**Table 1:** Core laboratory bifurcation dedicated quantitative coronary angiography analysis.

		Median	Interquartile range (Q1–Q3)
Baseline SYNTAX score		13	9–24

LM	RefD (mm)	4.17	3.7–4.7
	DS (%)	52	30–59
	Finet-RefD (mm)	4.11	3.8–4.5
	Finet-DS (%)	51	37–60
	MLD (mm)	2.03	1.7–2.5

LAD	RefD (mm)	3.09	2.8–3.4
	DS (%)	43	15–59
	MLD (mm)	1.72	1.3–2.5

LCx	RefD (mm)	2.92	2.6–3.2
	DS (%)	19	9–31
	MLD (mm)	2.43	1.8–2.8

Abbreviations: DS = diameter stenosis, LAD = left anterior descending artery, LCx = left circumflex, LM = left main, MLD = minimum lumen diameter, RefD = reference diameter.

**Table 2:** MEDINA classification of actual enrollment (highest %DS according to Bif-quantitative coronary angiography [QCA] or Finet-QCA) vs. MEDINA classification by visual assessment.

QCA-based MEDINA	Visual assessment MEDINA								
	1,1,1	1,1,0	1,0,1	1,0,0	0,1,1	0,1,0	0,0,1	0,0,0	Total
1,1,1	1	0	1	0	0	0	0	0	2
1,1,0	1	8	1	1	0	1	0	0	12
1,0,1	0	0	2	0	0	0	0	0	2
1,0,0	2	6	5	25	1	3	0	0	42
0,1,1	3	0	0	0	0	0	0	0	3
0,1,0	1	2	0	2	0	18	0	0	23
0,0,1	0	0	0	0	0	0	0	0	0
0,0,0	0	0	0	0	0	0	0	0	0
Total	8	16	9	28	1	22	0	0	84

*Note:* kappa = 0.510, agreement = 64%.

**Table 3:** MEDINA classification of Bif-quantitative coronary angiography (QCA) alone vs. MEDINA classification by visual assessment.

Bif-QCA (without Finet)	Visual assessment MEDINA								
	1,1,1	1,1,0	1,0,1	1,0,0	0,1,1	0,1,0	0,0,1	0,0,0	Total
1,1,1	1	0	0	0	0	0	0	0	1
1,1,0	1	8	1	1	0	1	0	0	12
1,0,1	0	0	2	0	0	0	0	0	2
1,0,0	2	6	4	23	1	3	0	0	39
0,1,1	3	0	1	0	0	0	0	0	4
0,1,0	1	2	0	2	0	18	0	0	23
0,0,1	0	0	1	0	0	0	0	0	1
0,0,0	0	0	0	2	0	0	0	0	2
Total	8	16	9	28	1	22	0	0	84

*Note:* kappa = 0.487, agreement = 62%.

**Table 4:** MEDINA classification of Finet-QCA alone vs. MEDINA classification by visual assessment.

Finet-QCA	Visual assessment MEDINA								
	1,1,1	1,1,0	1,0,1	1,0,0	0,1,1	0,1,0	0,0,1	0,0,0	Total
1,1,1	1	0	1	0	0	0	0	0	2
1,1,0	0	6	1	0	0	0	0	0	7
1,0,1	0	0	2	0	0	0	0	0	2
1,0,0	0	4	4	23	0	3	0	0	34
0,1,1	3	0	0	0	0	0	0	0	3
0,1,0	2	4	0	3	0	19	0	0	28
0,0,1	0	0	0	0	0	0	0	0	0
0,0,0	2	2	1	2	1	0	0	0	8
Total	8	16	9	28	1	22	0	0	84

*Note:* kappa = 0.481, agreement = 59%.

**Table 5:** MEDINA classification according to Bif-quantitative coronary angiography (QCA) and Finet-QCA.

Bif-QCA MEDINA (without Finet)	Finet-QCA MEDINA								
	1,1,1	1,1,0	1,0,1	1,0,0	0,1,1	0,1,0	0,0,1	0,0,0	Total
1,1,1	1	0	0	0	0	0	0	0	1
1,1,0	0	7	0	0	0	5	0	0	12
1,0,1	0	0	1	1	0	0	2	0	2
1,0,0	0	0	0	31	0	0	0	8	39
0,1,1	1	0	0	0	3	0	0	0	4
0,1,0	0	0	0	0	0	23	0	0	23
0,0,1	0	0	1	0	0	0	0	0	1
0,0,0	0	0	0	2	0	0	0	0	2
Total	2	7	2	34	3	28	8	8	84

*Note:* kappa = 0.696, agreement = 78.6%.

## Data Availability

The data that support the findings of this study are available from the corresponding author R.G., upon reasonable request.

## Nomenclature

CABG: Coronary artery bypass graft
%DS: Diameter stenosis (%)
LM: Unprotected left main
MI: Myocardial infarction
PCI: Percutaneous coronary intervention
QCA: Quantitative coronary angiography
RefD: Reference diameter
Bif-QCA: Bifurcation-dedicated quantitative coronary angiography
Finet-QCA: Finet's law–derived Bif-QCA
FFR: Fractional flow reserve
iFR: Instantaneous wave-free ratio
LAD: Left anterior descendent artery
LCx: Left circumflex artery.

## Supporting Information

Supporting Table 1: Baseline characteristics. Supporting Table 2: iFR details. Supporting Figure 1: ROC curves of Bif-QCA derived %DS (≥ 50) in predicting an abnormal iFR value (less or equal to 0.89). (a) Standard Bif-QCA %DS (AUC-ROC 0.535, 95% CI 0.35–0.72), (b) Finet-derived Bif-QCA %DS (AUC-ROC 0.444, 95% CI 0.28–0.61). Supporting Figure 2: Representative cases where differences in reference diameter and %DS between Bif-QCA and Finet-QCA are observed. The examples highlight how Finet-QCA derives the proximal reference diameter using daughter-vessel diameters, which may either over- or underestimate stenosis severity compared to the segmental analysis of Bif-QCA, particularly in the presence of diffuse disease of PMV (a) or vessel tapering/diffuse disease of DMV/SB (b).
