# Supplementary material for: Fractal Laws for Bifurcation Quantitative Coronary Angiography to Assess Left Main Bifurcation Lesions
Source: Cardiol Res Pract. 2025 Jun 1;2025:7176161. doi: 10.1155/crp/7176161 (PMC12145935; doi:10.1155/crp/7176161)

**Supplementary material**

**Supplementary Table 1.** Baseline characteristics

| Variables | Values |
| --- | --- |
| Age, years | 68.2±9.4 |
| BMI, kg/m^2^ | 28.0±3.9 |
| LVEF, % | 55.3±9.2 |
| Smoking status, n (%) |  |
| Never | 38 (45.2) |
| Current smoker | 13 (15.5) |
| Previous smoker | 33 (39.2) |
| Diabetes mellitus, n (%) | 29 (34.5) |
| Hypertension, n (%) | 67 (79.7) |
| Dyslipidemia, n (%) | 60 (71.4) |
| Previous myocardial infarction, n (%) | 15 (18.5) |
| Peripheral vascular disease, n (%) | 8 (9.5) |
| COPD, n (%) | 7 (8.3) |
| Heart failure, n (%) | 6 (7.1) |
| Previous major bleeding, n (%) | 2 (2.5) |
| Previous PCI, n (%) | 30 (35.7) |
| Previous CABG, n (%) | 1 (1.2) |
| CAD history, n (%) | 17 (20.2) |
| Clinical presentation, n (%) |  |
| Stable angina | 52 (61.9) |
| Unstable angina/NSTEMI | 11 (13.0) |
| Silent Ischemia | 21 (25.0) |
| Hemoglobin, g/dL | 13.5±2.2 |
| WBC, cells*10^9^L | 7.8±2.4 |
| Platelets, cells*10^9^L | 226.6±71.7 |
| Creatinine, mg/dL | 1.0±0.3 |
| HbA1c, % | 6.2±1.1 |

BMI: body mass index, LVEF: left ventricular ejection fraction, COPD: chronic obstructive pulmonary disease, PCI: percutaneous coronary intervention, CABG: coronary artery bypass graft, CAD: coronary artery disease, WBC: white blood cells.

**Supplementary Table 2.** iFR details.

|  | LAD (n=83) | LCx (n=55) | p.value |
| --- | --- | --- | --- |
| iFR value, median [IQR] | 0.80 [0.71-0.91] | 0.95 [0.90-1.00] | <0.001 |
| Positivity rate, n (%) | 70 (80.3) | 20 (36.4) | <0.001 |

IQR= interquatile range, LAD= left anterior descending, LCx= left circumflex

Differences between both groups were assessed with the use of Mann-Whitney U test.

**Supplementary Figure 1.** ROC curves of Bif-QCA derived %DS (≥50) in predicting an abnormal iFR value (less or equal to 0.89). a) standard Bif-QCA %DS (AUC-ROC 0.535, 95% CI 0.35-0.72), b) Finet-derived Bif-QCA %DS (AUC-ROC 0.444, 95% CI 0.28-0.61).


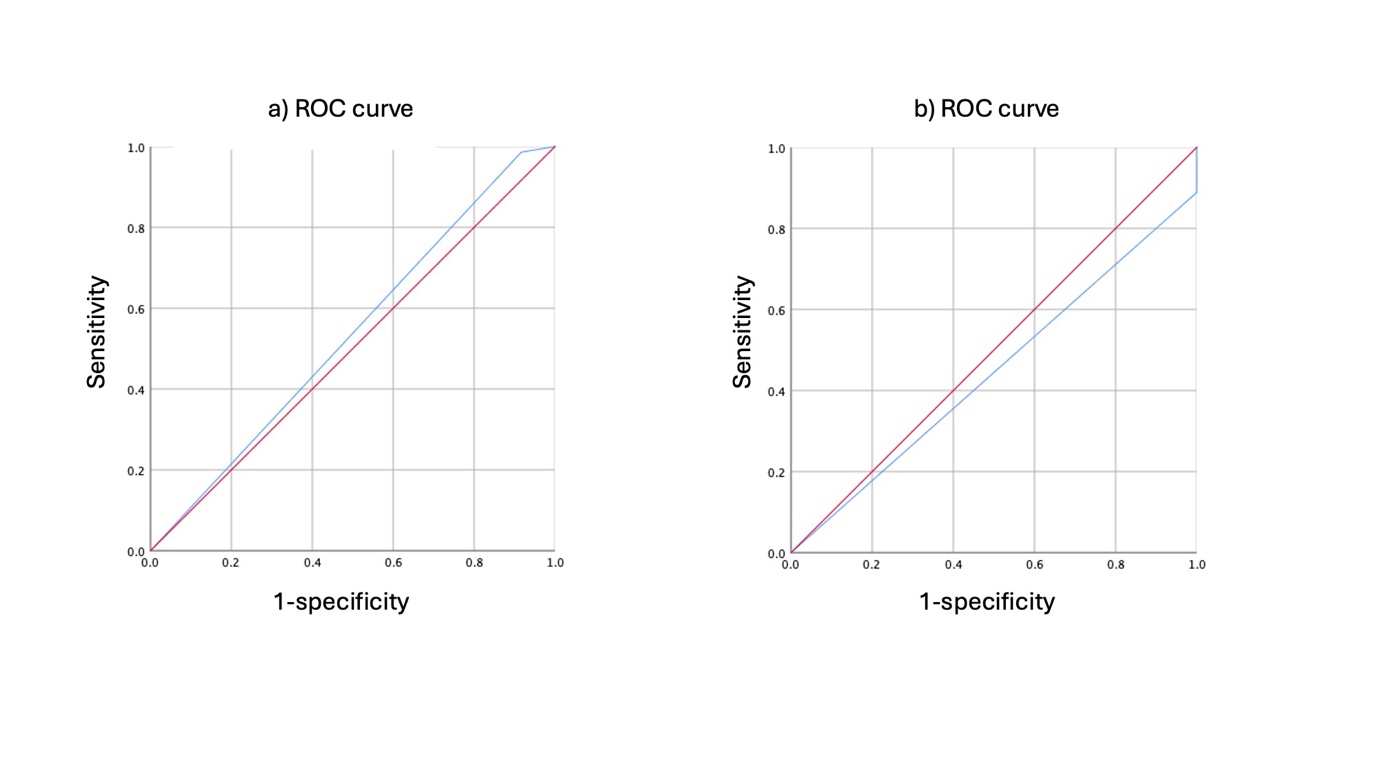


**Supplementary Figure 2.** Representative cases where differences in reference diameter and %DS between Bif-QCA and Finet-QCA are observed. The examples highlights how Finet-QCA derives the proximal reference diameter using daughter-vessel diameters, which may either over- or underestimate stenosis severity compared to the segmental analysis of Bif-QCA, particularly in presence of diffuse disease of PMV (a) or vessel tapering/diffuse disease of DMV/SB (b).


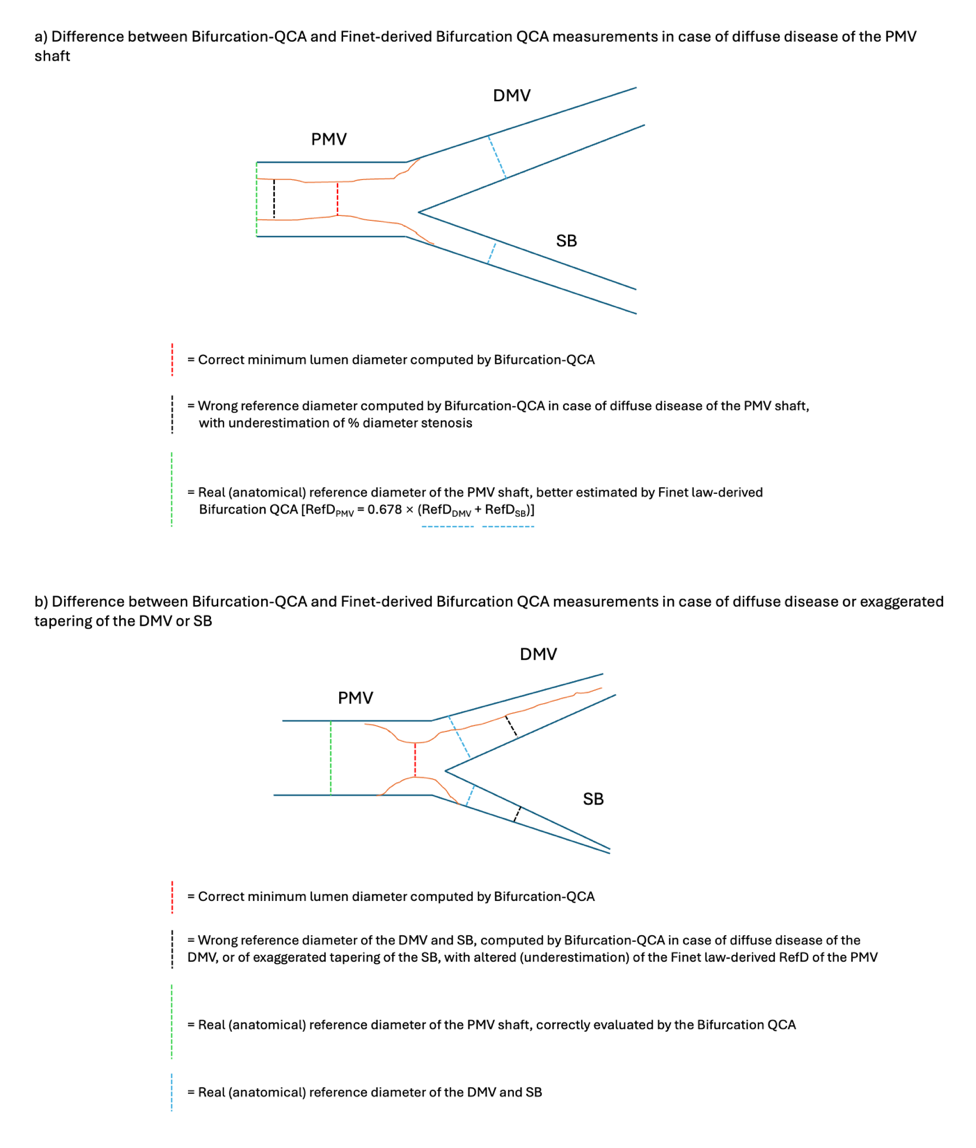

Supplement: Supporting Information — Additional supporting information can be found online in the Supporting Information section. [file 7176161.f1.docx]
